# Prognostic value of gastric cancer‐associated gene signatures: Evidence based on a meta‐analysis using integrated bioinformatics methods

**DOI:** 10.1111/jcmm.13823

**Published:** 2018-08-22

**Authors:** Jun Wang, Peng Gao, Yongxi Song, Jingxu Sun, Xiaowan Chen, Hong Yu, Yu Wang, Zhenning Wang

**Affiliations:** ^1^ Department of Surgical Oncology and General Surgery The First Hospital of China Medical University Heping District, Shenyang China

**Keywords:** gastric cancer, meta‐analysis, network mining, prognosis, robust rank aggregation

## Abstract

Selecting differentially expressed genes (DEGs) based on integrated bioinformatics analyses has been used in previous studies to explore potential biomarkers in gastric cancer (GC) with microarray and RNA sequencing data. However, the genes obtained may be inaccurate because of noisy data and errors, as well as insufficient clinical sample sizes. Thus, we aimed to find robust and strong DEGs with prognostic value for GC, where the robust rank aggregation method was employed to select significant DEGs from eight Gene Expression Omnibus data sets with a total of 140 up‐regulated and 206 down‐regulated genes. Network data mining was then used to screen hub genes, and 11 genes were filtered using Fisher's exact test. Based on these results, we built a prognostic signature with seven genes (*FBN1*,*MMP1*,*PLAU*,*SPARC*,*COL1A2*,*COL2A1* and *ATP4A*) using stepwise multivariate Cox proportional hazard regression. According to the risk score for each patient, we found that high‐risk group patients had significantly worse survival results compared with those in the low‐risk group (log‐rank test *P*‐value < 0.001). This seven‐gene signature was then validated with an external data set. Thus, we established a signature based on seven DEGs with prognostic value for GC patients using multi‐steps bioinformatics methods, which may provide novel insights and potential biomarkers for prognosis, as well as possibly serving as new therapeutic targets in clinical applications.

## 1 INTRODUCTION

A previous study estimated that 951 600 new GC cases and 723 100 deaths occurred in 2012, especially in Eastern Asia.^1^ Although a dramatic worldwide decline in the incidence and mortality rates of GC has occurred, GC still has a poor 5‐year survival rate.^2^ Therefore, molecular biomarkers have attracted much attention because of diagnosing and evaluating the prognosis in GC.

Microarray and RNA sequencing technologies, as well as gene profiling data sets such as The Cancer Genome Atlas (TCGA) and the Gene Expression Omnibus (GEO), have been used to identify various DEGs and significant biological pathways in different cancers. Several recent studies of DEGs associated with GC^3^, ^4^, ^5^ have employed integrated bioinformatics analyses to explore the patterns of gene expression. However, biased gene expression results may be obtained using a single data set because of data outliers, noise, and errors, as well as insufficient sample sizes. The robust rank aggregation (RRA) method has been employed for selecting differentially expressed microRNA (miRNA) profiles based on multiple data sets in various cancers, which is robust to these noises. However, no previous study of GC has identified DEGs using the RRA method, especially to detect prognostic gene signatures, which motivated this study.

In this study, we performed multi‐step analysis to examine prognostic gene signatures in order to determine whether the RRA method can be used for selecting DEGs from a variety of GEO data sets and for identifying prognostic biomarkers in GC.

## 2 MATERIALS AND METHODS

### 2.1 Gene expression omnibus data set selection and data generation

Eight independent GC gene expression microarray data sets were downloaded from the GEO database. A summary of the detailed series information is shown in Table S1. The filter of DEGs according to the criteria of: |log2 fold‐change| > 1 and adj.*P*‐value < 0.05.

### 2.2 Robust rank aggregation method for meta‐analysis

In order to avoid inconsistent results among different studies and to identify robust DEGs based on the GC data sets, the RRA method^6^ was applied to the lists of genes, which employs a probabilistic model for aggregation. The RRA method is robust to noise, and it facilitates the calculation of significance probabilities to all of the elements in the final ranking. The *P*‐values were subjected to Bonferroni's correction to avoid false‐positive results.

### 2.3 Network data mining from DEGs

To determine more accurate and robust DEGs with prognostic value in GC based on above genes we obtained. The PPI networks were built by the Human Integrated Protein‐Protein Interaction rEference (HIPPIE), and Fisher's exact test was used to select hub genes according to a *P*‐value cut‐off < 0.01 and mapping number > 5.

### 2.4 Prognostic gene signature risk scoring system based on DEGs

Stepwise multivariate Cox proportional hazard regression was performed to obtain the regression coefficient for each gene. The area under the time‐dependent receiver operating characteristic (ROC) curve (AUC) was determined to predict the 5‐year survival, and high‐ and low‐risk groups were according to the median‐risk score. The Kaplan‐Meier curve was plotted to compare the survival outcomes in different groups. Gene Expression Profiling Interactive Analysis (GEPIA)^7^ is a web server that used to analyse the gene expression patterns in different TNM stages of GC.

## 3 RESULTS

**Figure 1:**
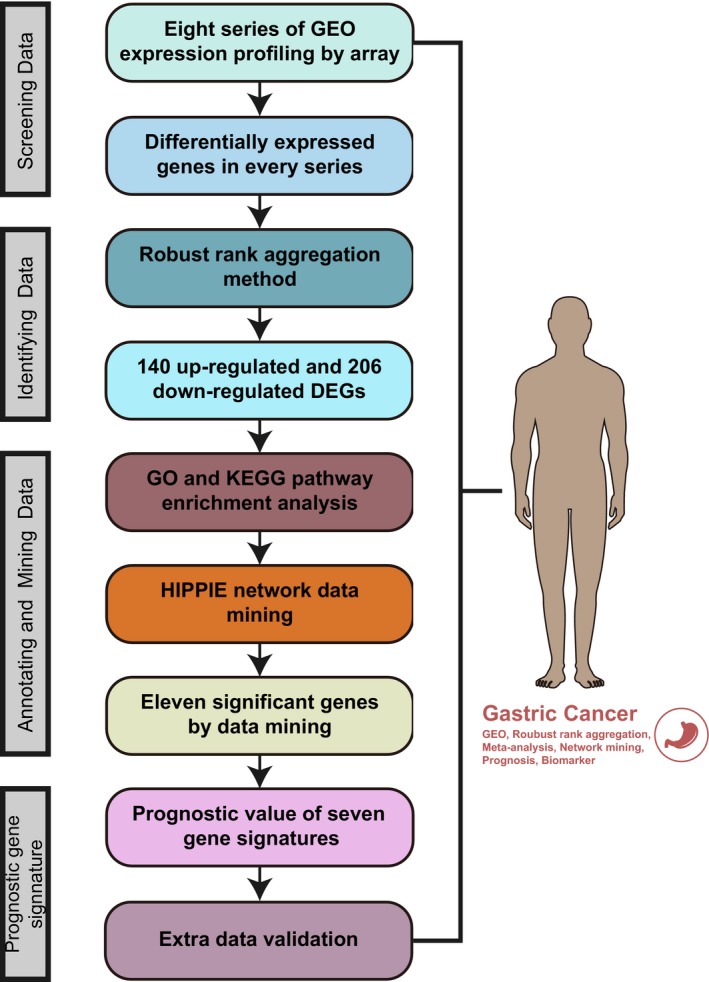
Workflow of our multi‐step strategy for identifying a gene signature with prognostic value in gastric cancer (GC)

In this study, we employed a multi‐step strategy to obtain a signature for DEGs with prognostic value in GC patients (Figure 1). We first downloaded eight GEO data sets of GC with 493 tumour and 213 normal samples. Significant DEGs were then filtered out from each GEO data set (Table S2). The RRA method^6^ was next applied to screen out precise and robust DEGs with 140 significantly up‐regulated and 206 down‐regulated (Table S3). GO processes and pathways enrichment results are shown in Figure S1.

Based on above method, we have obtained plentiful DEGs of GC. However, these genes may not all play significant biological roles. To find robust and strong DEGs from above genes, we combined various network data mining methods. The PPI networks were built (Figure S2A) based on a confidence score >0.6. Fisher's exact test (Table S5) was used to select hub genes according to a *P*‐value < 0.01 and mapping number > 5. Therefore, 11 hub genes were filtered (Table S6).

**Figure 2:**
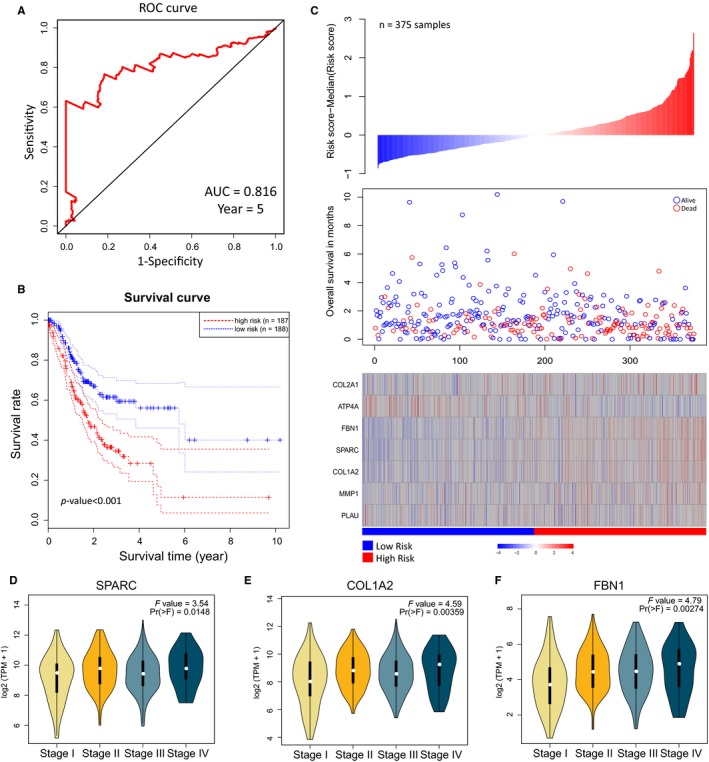
Establishment of a seven‐gene signature prognostic risk scoring system based on above DEGs. A, Time‐dependent ROC curve for predicting the 5‐y survival. B, Kaplan‐Meier curve for the seven‐gene signature (log‐rank test *P*‐value < 0.001). The two dotted lines in each group are the level for a two‐sided confidence interval on the survival curve. C, The seven‐gene signature‐based risk score distributions, patient survival results and expression heatmap. D‐F, Expression profiles of *SPARC*,*COL1A2* and *FBN1* in different TNM stages of GC

However, to find key DEGs with prognostic role in GC, we next built a prognostic signature with seven genes (*FBN1*,* MMP1*,* PLAU*,* SPARC*,* COL1A2*,* COL2A1* and *ATP4A*) using stepwise multivariate Cox proportional hazard regression. The AUC was 0.816 for predicting the 5‐year survival (Figure 2A). According to the median risk score of each patient, we found that the patients from the high‐risk group had significantly poorer overall survival results compared with those in the low‐risk group (log‐rank test *P*‐value < 0.001) (Figure 2B). The seven‐gene signature risk score distributions, patient survival results and expression heatmap are shown in Figure 2C. *SPARC*,* COL1A2* and *FBN1* were differentially expressed in various TNM stages of GC (Figure 2D‐F).

Finally, this seven‐gene signature was then validated with an independent data set (GSE62254, n = 300 samples) (Figure S4A,B). The Kaplan‐Meier curves indicated that there were significant differences between the high‐ and low‐risk groups (log‐rank test *P*‐value < 0.001). Therefore, this seven‐gene signature may be useful for prediction of the survival and prognosis of GC.

## 4 DISCUSSION

Molecular biomarkers such as genes have attracted much attention because they can be useful for the pathogenesis of GC. Thus, several recent studies have investigated the DEGs in GC. One study has reported that using 26 paired GC samples and microarray analysis, 2371 differential mRNAs were detected.^5^ In addition, several studies have investigated prognostic gene signatures in GC. A group expression signature based on five genes was established using univariate survival analysis and the LASSO method.^8^ However, there have been no previous reports of the detection of DEGs in GC using the RRA method.

In this study, we not only selected significant DEGs using integrated and robust bioinformatics methods comprising various GEO series, the RRA method and network data mining. But also we developed a seven‐gene signature with prognostic value. The RRA method can avoid results error of a mass of DEGs we obtained. In our network data mining, we used HIPPIE and Fisher's exact test to generate significant hub genes, which were more accurate and robust. Moreover, based on above DEGs, we establish a seven‐gene signature which was validated with an external data set independently and accurately.

As for these seven genes, six of them play important roles in the molecular mechanism of GC progression. *SPARC* has been shown associated with cancer progression.^9^ Moreover, *SPARC,* 10 *COL1A2* 11 and *ATP4A* 12 were identified as DEGs in GC, which were consistent with our results. *PLAU* was involved in the prediction of GC patient survival^13^ and *MMP1* is mostly associated with genetic polymorphisms.^14^, ^15^ However, no previous study has considered the possible role of *FBN1* in GC.

In this study, we not only find robust and strong DGEs in GC using integrated multi‐step analysis including RRA method, enrichment analysis and network data mining, but also build a seven‐gene signature with prognostic value for GC based on above DEGs. Our findings may provide novel insights and potential biomarkers for GC prognosis.

## CONFLICTS OF INTEREST

The authors declare that they have no conflicts of interest.
