# Supplementary material for: Prognostic value of gastric cancer‐associated gene signatures: Evidence based on a meta‐analysis using integrated bioinformatics methods
Source: J Cell Mol Med. 2018 Aug 22;22(11):5743–7. doi: 10.1111/jcmm.13823 (PMC6201382; doi:10.1111/jcmm.13823)
Supplement: Supplementary file 11 [file JCMM-22-5743-s011.docx]

**Supplementary Information**

**Supplementary Materials and methods**

**Statistical analyses**

The Mann–Whitney U test was employed to compare the DEGs in GC and normal tissues. Fisher’s exact test was performed to explore significant hub genes according to a *p*-value < 0.01. The log-rank test was applied to compare the outcomes of the Kaplan–Meier curve analysis. A two-tailed *p*-value < 0.05 was considered to indicate a significant difference.

**Supplementary Results**

**Establishment of a seven-gene signature prognostic risk scoring system**

We next built a prognostic signature with seven genes (*FBN1, MMP1, PLAU, SPARC, COL1A2, COL2A1*, and *ATP4A*) using stepwise multivariate Cox proportional hazard regression. First, we used the Cox proportional hazard regression to obtain the regression coefficient of each gene, using the Survival package in the R. Second, we established the risk scoring system based on the individual expression values (log2-transformed) as follows: risk score = (0.3075) * *FBN1* value + (0.0921)* *MMP1* value + (-0.1594)* *PLAU* value + (0.5315)* *SPARC* value + (-0.4617)* *COL1A2* value + (0.0677)* *COL2A1* value + (-0.0774)* *ATP4A* value. Next, we obtained risk score of all patients one by one in the training or validation data set. Then, the median risk score was the one who ranks in the middle location of all risk scores. The median risk score in the training set was 1.0508, besides, the one in the validation set was 0.8320. Finally, we separated all patients into high- and low-risk groups according to the median risk score.

**Supplementary Tables Legends**

**Supplementary Table S1.** The summary of eight GEO data sets with gene expression profiles.

**Supplementary Table S2.** Differentially expressed genes screening in each GEO data sets.

**Supplementary Table S3.** Differentially expressed genes screening based on RRA method.

**Supplementary Table S4.** GO processes and KEGG pathways enrichment analyses of DEGs.

**Supplementary Table S5.** PPI and Fisher’s exact test analyses of all DEGs.

**Supplementary Table S6.** Hub genes selection based on PPI and Fisher’s exact test.

**Supplementary Figures Legends**

**Supplementary Figure S1. Characteristic molecular annotations of DEGs.** (A) Heatmap of the top ten significantly up- and downregulated DEGs in eight GEO data sets. (B) GO term enrichment analysis of upregulated genes. (C) GO term enrichment analysis of downregulated genes. (D) KEGG pathway enrichment analysis of upregulated genes. (E) KEGG pathway enrichment analysis of downregulated genes.

**Supplementary Figure S2. Eleven hub genes selected by network data mining.** (A) Protein–protein interaction networks with confidence scores > 0.6 in the HIPPIE database. (B) Enriched pathways identified based on the 11 hub genes. (C) Relationships determined among the enriched pathways using the *clusterProfiler* R package. (D) Violin plots of the expression profiles of 11 genes from the TCGA database.

**Supplementary Figure S3. Visual KEGG pathway analysis with the gene expression profiles based on the pathway enrichment results**.

**Supplementary Figure S4. Validation of the seven-gene signature for predicting survival in GC.** (A) Expression profiles of the seven genes in the GSE62254 data set. (B) Kaplan–Meier curves for the seven-gene signature in the validation set (log-rank test *p*-value < 0.001). (C) Pearson’s correlation coefficients of the seven genes in the GSE62254 data set. (D) Pearson’s correlation coefficients of the seven genes in the TCGA data set.
